# Efficacy of jianpi huatan granule in reducing colorectal cancer metastasis and recurrence after radical resection and adjuvant chemotherapy: Study protocol for a randomised, double-blind, placebo-controlled, multicentre trial

**DOI:** 10.3389/fphar.2022.944475

**Published:** 2022-09-13

**Authors:** Liusheng Li, Qian Qu, Ning Cui, Linlin Cai, Jianhua Zou, Jiao Wu, Tengteng Hao, Yu Wu

**Affiliations:** ^1^ Department of Oncology, Xiyuan Hospital, Chinese Academy of Traditional Chinese Medicine, Beijing, China; ^2^ College of Integrated Traditional Chinese and Western Medicine, Binzhou Medical University, Binzhou, China

**Keywords:** jianpi huatan granule, metastasis and recurrence, traditional Chinese medicine, randomized controlled trial, protocol, stage III and high-risk stage II colorectal cancer

## Abstract

**Background:** The high incidence and mortality rates of colorectal cancer (CRC) are a severe challenge in China. In patients with stage III and high-risk stage II CRC after radical resection and postoperative adjuvant chemoradiotherapy, 40–60% experience recurrence and metastasis. Several years of clinical practice have shown that traditional Chinese medicine, including Jianpi Huatan granule (JHG), effectively prevents stage III and high-risk stage II CRC recurrence and metastasis after radical resection and postoperative standard adjuvant chemotherapy. However, high-level systematic plans and evidence-based medicine are lacking in this regard. Therefore, this randomised control trial aimes to determine the efficacy of JHG in reducing stage III and high-risk stage II CRC metastasis and recurrence after radical resection and postoperative standard adjuvant chemotherapy.

**Methods:** This is a multicentre, randomised, double-blind, placebo-controlled clinical trial. Three hundred and fifty patients with stage III or high-risk stage II CRC who completed adjuvant chemotherapy after radical resection will be recruited from eight medical centres in China and randomly assigned to test (n = 175) and control (n = 175) groups at a ratio of 1:1. The test group will receive oral JHG for 3 months, whereas the control group will receive oral placebo for 3 months. The primary outcomes will be the disease-free survival and 1-, 2-, and 3-years metastasis and recurrence rates, whereas the secondary outcomes will be quality of life and circulating tumour cells. The patients will be followed-up monthly during treatment and every 3–6 months thereafter until recurrence, metastasis, death, or the end of the study.

**Trial registration:** This trial was registered at ClinicalTrials.gov (NCT03716063).

## 1 Introduction

Colorectal cancer (CRC) is one of the most common malignant tumours worldwide. A global epidemiological study of cancer showed that CRC ranks third in incidence and second in mortality resulting from tumour malignancy (Sung et al., 2021). Moreover, its annual incidence is increasing, which seriously threatens human life and health (Siegel et al., 2019). According to the 2018 global cancer statistics (Bray et al., 2018), there were an estimated 18.1 million new cancer cases and 9.6 million cancer deaths in 2018 worldwide. The annual incidence and mortality rates of CRC have recently decreased in Western countries with improvements in early cancer prevention and treatment (Siegel et al., 2018). However, in China, the incidence of CRC has been increasing annually owing to its aging population, industrialisation, and westernised diet (Li and Gu, 2005; Winkels et al., 2021). Cancer Analysis (2015) released in 2016 (Miller et al., 2016) showed that the incidence of gastrointestinal malignant tumours in China was 1,055.4 per 100,000, with a mortality rate of 689.0 per 100,000 individuals. The incidence and mortality rates of CRC account for 25% of all CRC cases. Therefore, it is essential to develop effective preventive measures and treatments for CRC.

In patients with stage III and high-risk stage II CRC, 40–60% experience recurrence, metastasis, and progression to advanced-stage disease after radical surgery and postoperative adjuvant chemoradiotherapy (Chen et al., 2016). At present, there is no internationally recognized drug for preventing metastasis and recurrence. Therefore, preventing the recurrence and metastasis of early stage CRC is essential (Wang et al., 2021). In China, traditional Chinese medicine (TCM) is widely used to treat CRC, particularly to reduce the recurrence and metastasis rates of postoperative CRC (Li and Chi, 2011; Sun et al., 2021). Clinical trials have shown that TCM achieves good results in the treatment of CRC, especially in reducing metastasis and recurrence of postoperative CRC (Pita-Fernández et al., 2015). However, most of the previous studies conducted were cohort studies (Xu et al., 2017; Yang et al., 2008); therefore, large-sample, multicentre, randomised controlled trials are needed to provide high-level evidence of the efficacy of TCM in the treatment of CRC.

Jianpi Huatan granule (JHG) is prescribed in TCM and are widely used for the treatment of CRC in clinical practice. Our research group condensed this prescription according to several clinical studies (Xu et al., 2017; Chen et al., 2019), and we hope to explore its clinical efficacy and safety more rigorously. Therefore, this multicentre, large-sample, randomised controlled trial will evaluate the efficacy of JHG in reducing stage III and high-risk stage II CRC metastases and recurrence rates after radical resection and standard adjuvant chemotherapy.

## 2 Materials and methods

### 2.1 Trial objectives and design

**FIGURE 1:**
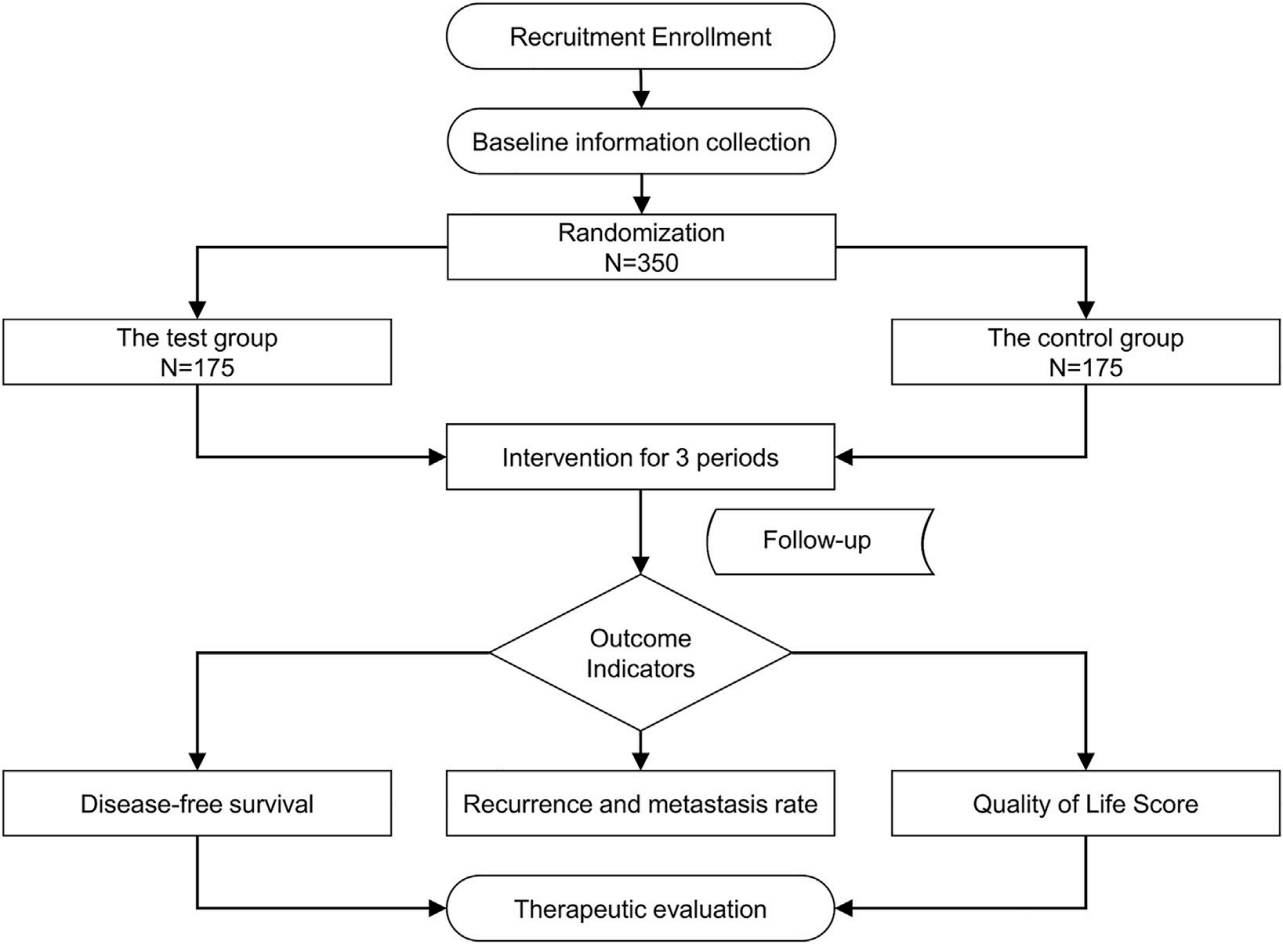
Flow chart of the study procedures.

To evaluate the clinical efficacy of JHG in patients with stage III and high-risk stage II CRC who completed adjuvant chemotherapy after radical resection, a large-sample, multicentre, randomised, double-blind, placebo-controlled clinical trial will be conducted. Three hundred and fifty participants with stage III or high-risk stage II CRC who completed adjuvant chemotherapy after radical resection will be recruited. In- and outpatients will be recruited from eight centres (Xiyuan Hospital of China Academy of Chinese Medical Sciences, Cancer Hospital of Chinese Academy of Medical Sciences, Tianjin People’s Hospital, Beijing Chaoyang Hospital of Capital Medical University, Henan Cancer Hospital, Nanjing Hospital of TCM, Peking University First Hospital, and First Affiliated Hospital of Guiyang College of TCM). The study flow is summarised in Figure 1.

### 2.2 Randomisation and blinding

A central stochastic approach will be followed. A random sequence will be generated by professional statisticians using the SAS (PROC PLAN) statistical software (SAS Institute Inc., Cary, NC, United States). Three hundred and fifty participants will be divided into test and control groups at a ratio of 1:1, with 175 cases in each group. Random seeds and sequences will be stored confidentially in opaque envelopes, and emergency unblinded envelopes will be submitted to a third party to ensure that patients, researchers, and outcome evaluators remain unaware of patient allocations. After successful screening and signing of the informed consent form, the researcher will open a random envelope and record each participant’s name, hospital number, and admission time^1^. According to the randomized results, the test group will receive JHG, whereas the control group will receive placebo (resembling JHG in appearance, color, clarity, odor and taste).

### 2.3 Participants recruitment

#### 2.3.1 Inclusion criteria

1) Clear pathological diagnosis of CRC that conforms to Western medicine diagnostic criteria;
2) Radical resection of CRC was performed, and at least 3 months of standard adjuvant chemotherapy administered before starting the trial, including preoperative neoadjuvant therapy for rectal cancer;
3) TNM stage III or high-risk stage II;
4) Age 18–80 years for both sexes;
5) No recurrence or metastasis by imaging or doctor’s clinical judgment; and
6) Provision of written informed consent.

#### 2.3.2 Exclusion criteria

1) History of previous or combined malignancies except cured basal cell carcinoma of the skin and carcinoma *in situ* of the cervix;
2) Presence of severe heart, liver, or kidney disease;
3) Any unstable condition that may endanger patient safety and compliance with research, such as pregnancy, depression, manic–depressive disorder, obsessive–compulsive disorder, or schizophrenia; and
4) Researcher determination that a patient is unsuitable for the study.

#### 2.3.3 Dropout criteria

1) All participants who signed the informed consent form and will be screened for trial eligibility will be referred to as exfoliated cases whenever and for any reason they withdrew from the study and failed to complete the observation period stipulated in the plan;
2) When a participant falls behind, the researcher will contact them as much as possible to request reasons and record the last medication time to complete the evaluation project; and
3) When participants withdraw owing to adverse events or ineffective treatment, the researchers will take appropriate treatment measures according to the actual participant’s situation.

#### 2.3.4 Suspension/exit criteria

1) The researchers may decide to end/suspend patient participation when the participant appears unsuitable to continue. For all cases of trial termination, the withdrawal reasons and dates will be recorded, and the reasons will be statistically analysed at the end of the study; and
2) The following will be the criteria for terminating a case:
  a) Severe adverse events (life-threatening or impaired normal work and life) which will prevent treatment continuation;
  b) Severe, persistent allergic reactions that cause treatment discontinuation, in which case the treatment will be considered ineffective;
  c) Serious violation of the experimental plan;
  d) Absence or withdrawal from the trial;
  e) Endpoint of observation (tumour recurrence or metastasis) or inability to take Chinese herbal medicine orally; and
  f) Other.

### 2.4 Interventions

1) The test group will receive oral JHG in the morning and evening for 3 months. JHG will consist of 30 g of *Astragalus mongholicus* Bunge [Fabaceae; Astragali radix], 10 g of *Panax ginseng* C. A. Mey. [Araliaceae; Ginseng radix et Rhizoma], 30 g of *Poria cocos* (Schw.) Wolf [Polyporaceae; Poria fungus nucleus], 10 g *Epimedium brevicornu* Maxim. [Berberidaceae; Epimedii folium], and 6 g of *Arisaema erubescens* (Wall.) Schott [Araceae; Arisaematis rhizoma preparatum]. JHG will be prepared as follows: five ingredients will be decocted twice with 3-fold distilled water according to the above ratio at each course for 1 h. The decocting solution will then be combined. The supernatant obtained by high-speed centrifugation will be concentrated at 50°C to a relative density of 1.08–1.10 g/ml. Subsequently, the same amount of 95% ethanol will be added, stirred and left to stand for 24 h. The supernatant will be filtered and the ethanol recovered, then further concentrated at 50°C to a relative density of 1.30–1.32 g/ml. Dextrin will be added and the solution will be granulated in a fluidised bed granulator. The obtained particles will be dried under vacuum at 50 °C for 1 h, packaged, and stored at 4°C.
2) The control group will receive oral placebo (mostly dextrin containing 10% of the dosage of JHG determined by appearance, color, clarity, odor and taste through several experiments) in the morning and evening for 3 months. The preparation process of the placebo will be the same as that of JHG.

JHG and placebo will be provided by Beijing Temages Pharmaceutical Co., LTD. (Beijing, China). The characteristics, particle size, moisture content, volume difference, and solubility of the JHG and placebo will be examined according to the granule section of the Pharmacopoeia of the People’s Republic of China (2020 edition).

### 2.5 Combined medications and treatments

All participants will be required to adhere to the following guidelines for combination medication and treatment during the trial period:

#### 2.5.1 Combined medications

1) Participants will be advised not to take herbal medicines containing *Epimedium* or *Arisaema* species.
2) Other drugs that must be continued when patients with other diseases are enrolled should be recorded in the medical record report form along with the participant’s other diseases and medication history.
3) Researchers will ask the participants to bring all the medications they were using at the hospital during the interview to check for their combinatory effects. Drugs or other therapies that must be continued must be documented in medical records, including dosage, frequency, and time of use for analysis and reporting.

#### 2.5.2 Combined treatments

Participants will be allowed to receive the necessary treatments for other illnesses or symptoms (unrelated to this indication); however, the treatment should be accurately and carefully documented in the study records.

### 2.6 Outcomes

#### 2.6.1 Primary outcomes

1) Disease-free survival will be defined as the time from the date of randomisation to the date of recurrence, metastasis, or death from any cause.
2) Recurrence and metastasis rates at 1, 2, and 3 years.

#### 2.6.2 Secondary outcomes

1) Quality of life score will be measured as follows:
  a) TCM symptom grade (Appendix 1) with the evaluation standards listed in Table 1.
  b) Functional Assessment of Cancer Therapy-Colorectal (FACT-C) (Version 4) scale (Appendix 2)*.* The FACT-C scale is divided into five grades: not at all (0), slightly (1), somewhat (2), quite a bit (3), and very much (4). Positive items (i.e. the higher the grade, the better the quality of life) will be directly scored from 0 to 4, whereas negative items will be reverse-scored. Items 1–7, 15, 17–20, 28, 29, 32, 35, and 36 are negative items. The scoring formula is as follows: forward item score = (0 + answer option number); reverse item score = (4 − answer option number). If there will be any unanswered questions, the score will be calculated by adding the scores of the answered questions to the total number of questions answered. The scores of all the fields will be summed to obtain the total score.
  c) Edmonton Symptom Assessment Scale (ESAS) (Appendix 3)*.* The scale uses a digital scoring method, with each symptom scored between 0 (asymptomatic) and 10 (most serious). Patients will choose a number to express their feelings, with a higher number indicating more serious symptoms. The points will be divided into three degrees: mild, 1–3; moderate, 4–6; and severe, 7–10.
2) Circulating tumour cells (CTCs) will be counted in the peripheral blood after randomisation and 3 months later.

**TABLE 1:** Treatment efficacy according to TCM Symptom Scale Score.

Effect level	Definition
Significantly effective	TCM Symptom Scale score decreased by >50% after versus before treatment
Effective	TCM Symptom Scale score decreased by 30–50% after versus before treatment
Ineffective	TCM Symptom Scale score decreased by <30% after versus before treatment

TCM, traditional Chinese medicine.

#### 2.6.3 Safety evaluation

1) All adverse events, including symptoms and signs.
2) General physical examination (height, weight, body temperature, blood pressure, heart rate, etc.) and essential physical examination (endocrine system and other special clinical parameters) data.
3) Laboratory examinations including routine blood tests, liver and kidney function tests, stool and occult blood tests, urine tests, and 12-lead electrocardiography.

### 2.7 Follow-up

#### 2.7.1 Follow-up time

The patients will be followed-up monthly during treatment and every 3–6 months thereafter until recurrence, metastasis, death, or the end of the study.

#### 2.7.2 Follow-up measurements/observations

##### 2.7.2.1 During treatment

1) Baseline physical examination parameters will include height, weight, blood pressure, and heart rate.
2) The safety indicators for each follow-up will include blood, urine, routine, stool, liver, and kidney function, and electrocardiography.
3) Imaging examination data of patients will be collected and recorded during treatment.
4) The participants in the group will be tested for CTCs after enrolment and again after treatment completion.
5) Symptom-based assessment will include the quality of life scale.

##### 2.7.2.2 After treatment

Follow-up observations will focus on metastasis and recurrence.

#### 2.7.3 Follow-up method

Patients will mainly be followed-up at outpatient clinics during treatment and by telephone until the observation deadline or endpoint events occur. The observation deadline will be the last day of contact.

### 2.8 Data management and monitoring

All data will be obtained from the medical records completed by the researcher. Data will be collected using an electronic case report form. The data manager is responsible for managing data and establishing a dedicated database for the project. Data entry will be performed by trained personnel, who will subsequently ensure data quality and accuracy. To ensure data accuracy, two data administrators will independently perform double entries and proofreading. Table 2 presents the details of the data-collection process.

**TABLE 2:** Study data collection process.

Study period											
Research stage	Enrolment	Intervention			Follow-up						
Visit number	1st	2nd	3rd	4th	5th	6th	7th	8th	9th	10th	11th
**Time point**	**Day 0**	**Day 30**	**Day 60**	**Day 90**	**Month 6**	**Month 9**	**Month 12**	**Month 18**	**Month 24**	**Month 30**	**Month 36**
Baseline basic information collected											
Eligibility screen	✔										
Informed consent	✔										
Demographic information	✔										
Medical and treatment history	✔										
Combined disease	✔	✔	✔	✔	✔	✔	✔	✔	✔	✔	✔
Combined medication	✔	✔	✔	✔	✔	✔	✔	✔	✔	✔	✔
Observation indices											
Recurrence and metastasis	✔	✔	✔	✔	✔	✔	✔	✔	✔	✔	✔
CTCs	✔			✔							
Vital signs	✔	✔	✔	✔							
Adverse reactions	✔	✔	✔	✔							
TCM Symptom Scale	✔	✔	✔	✔							
FACT-C	✔	✔	✔	✔							
ESAS	✔	✔	✔	✔							
Tumour markers	✔	✔	✔	✔							
Imaging examination	✔	✔	✔	✔	✔	✔	✔	✔	✔	✔	✔
Safety observations											
Routine blood tests	✔	✔	✔	✔							
Blood biochemistry tests	✔	✔	✔	✔							
Routine urine and stool test	✔	✔	✔	✔							
ECG	✔	✔	✔	✔							
Adverse events	✔	✔	✔	✔							

CTCs, circulating tumour cells; ECG, electrocardiography; ESAS, edmonton symptom assessment scale; FACT-C, functional assessment of cancer therapy-colorectal; TCM, traditional Chinese medicine.

### 2.9 Sample size calculation

The sample size was estimated using the PASS software (version 11; NCSS, LLC, Kaysville, UT, United States) and a log-rank test. The final sample size depends on several factors, including the occurrence of primary endpoint events.

The main endpoints of this study will be the postoperative recurrence and metastasis of stage II and III CRC. In previous studies (Xu et al., 2017; Yang et al., 2008), the 3-years recurrence and metastasis rates in the treatment and control groups were 18 and 31%, respectively. In this study, we used a 1:1 distribution design and an 80% test efficiency. The false-positive error rate will be controlled within 5% (both sides), and the annual loss rate in the follow-up period will be controlled within 5%. Based on the above hypothesis, 342 patients (171 and 171 in the experimental and control groups, respectively) were enrolled after a 1-year recruitment period and 3-years follow-up period. The number of expected endpoint events is 88 (33 in the experimental group and 55 in the control group). A minimum of 342 patients is required. This study will enrol 350 patients (175 in each group).

### 2.10 Statistical analysis

Statistical analyses will be performed using the SAS statistical analysis software (version 9.4; SAS Institute Inc., Cary, NC, United States). Data will analysed at the end of the study.

#### 2.10.1 Selection of analytical data sets

1) *Full analysis set:* The ideal set of participants will match the principle of intentionality analysis (mainly including all randomised participants) as closely as possible. The dataset was obtained by eliminating all randomised participants using the smallest and most reasonable method.
2) *Compliance scheme set:* In compliance with the experimental treatment scheme, the main variables can be measured, baseline variables will not be missing, and no major violations of the experimental scheme will be observed.
3) *Safety set:* All participants will receive at least one treatment after randomisation.
4) The full analysis set and compliance scheme set will be subjected to curative effect analysis, whereas the full analysis set will be used to analyse demographic and other baseline characteristics and therapeutic indicators.

#### 2.10.2 Contents of statistical analysis

The statistical analysis will include the actual number of participants enrolled in the two groups, dropouts, excluded cases, demographic and other baseline characteristics, compliance, efficacy, and safety analyses.

#### 2.10.3 Statistical analysis method

1) Descriptive statistical analysis: Qualitative indicators will be described using frequency tables, percentages, or constituent ratios, while quantitative indicators will be described using mean and standard deviation or median, lower quartile (Q1), upper quartile (Q3), minimum, and maximum.
2) Two groups of comparative analyses will be conducted. For qualitative data, the chi-square test, Fisher’s exact probability method, Wilcoxon rank-sum test, Cochran–Mantel–Haenszel-2 test, and weighted least-squares covariance test will be used. Quantitative data will be tested for normal distribution using a *t*-test (homogeneity test of variance between groups with a test level of 0.05, and the Satterthwaite method for correction of the *t*-test results when the variance is uneven). The Wilcoxon rank-sum test will be used in cases of non-conformity to the normal distribution, Kaplan–Meier survival analyses will be performed, and the Cox proportional risk model will be used for survival data. The hypothesis test uses a bilateral test and generates test statistics and corresponding P values. The test level will be set at 0.05, and statistical significance will be set at P < 0.05.

## 3 Discussion

The current treatment of stage II and III CRC involves a combination of surgery, radiotherapy, and chemotherapy (Munro et al., 2015; Snelgrove et al., 2015). Prognosis is determined according to clinical stage and risk factors (Oh et al., 2019). Patients are identified as incurable once the tumour recurs and metastasizes resulting in the death of nearly all patients (Ohlsson and Pålsson, 2003; Primrose et al., 2011). For tumours with high mortality rates, it is necessary to shift the focus from diagnosis and treatment to prevention. TCM has been widely used as a medical tool in China for cancer treatment. In previous studies, approximately 60–70% of patients with cancer in China received TCM (Sun et al., 2017; Sun et al., 2018). TCM treatment is also considered part of a healthy lifestyle to improve postoperative survival (Ching and Mok, 2022). Our research team has conducted a series of studies to prove the efficacy and safety of TCM in CRC treatment (Guo et al., 2012; Xu et al., 2017). TCM practitioners have also verified its safety and efficacy for cancer prevention and treatment in clinical practice (Liu et al., 2018; Lv et al., 2019).

To improve the clinical efficacy of TCM in the treatment of CRC and promote its use, further research into disease and syndrome combinations according to the main pathogenesis of CRC using strict multicentre clinical evaluations is necessary. In this study, patients with stage III CRC and high-risk stage II CRC after radical surgery and standard adjuvant chemotherapy will be selected as primary research participants. This large-sample, multicentre, double-blind, randomised controlled trial aims to demonstrate the efficacy of TCM in treating postoperative CRC by reducing metastasis and recurrence rates and improving patient quality of life.

CRC recurrence in patients with stages II and III CRC is currently being studied by considering its recurrence mechanism and prognostic factors. Patients with stage II and III CRC who were treated with adjuvant chemotherapy had a 25% survival benefit. In personalised medicine, it is necessary to identify CRC patients who may benefit from adjuvant chemotherapy (Cheng et al., 2018). Molecular diagnostics can guide treatment decisions for these patients (Kugimiya et al., 2019; Ntavatzikos et al., 2019). Regarding tumour recurrence–related factors, an increasing number of tumour markers can significantly predict the prognosis in stage II and III CRC cases (Lino-Silva et al., 2019). In addition, these tumour markers have a direct impact on the metastasis, recurrence status, and survival time of patients with stage II and III CRC (You et al., 2019). Moreover, researchers have developed CTC-based prognostic models to predict tumour recurrence in stage II and III CRC, which can be used to identify patients at high risk of recurrence and guide aggressive treatment to improve clinical outcomes (Rothé et al., 2019; Wang et al., 2019). In this study, we dynamically observed changes in CTCs in patients after radical surgery and chemotherapy, which is a new exploration and we hope to provide a better reference for the prognosis of patients with CRC.

In conclusion, this trial will determine the efficacy and safety of JHG in preventing metastasis and recurrence of stage III CRC and high-risk stage II CRC after radical surgery and standard adjuvant chemotherapy. Changes in the patient’s quality of life during the treatment period will be monitored. Monitoring CTCs in patients before and after treatment will provide new information regarding the tumour inhibition properties of TCM. Another objective of this study is to investigate the relationship between metastasis/recurrence and CTCs.

## Footnotes

1 TNM staging standard recommended by the American Joint Committee on Cancer and Union for International Cancer Control. T, Tumour; N, Node; M, Metastasis. According to the National Comprehensive Cancer Network Clinical Practice Guidelines in Oncology (2021 V.2 edition), stage II: T3–4N0M0. High-risk factors for stage II include: a. T4; b. Fewer than 12 lymph nodes are detected; c. Preoperative intestinal obstruction or perforation at the tumour site; d. poor histological differentiation (except highly unstable microsatellites); E. nerve invasion and vascular cancer thrombus; f. Positive or unknown margin, insufficient margin safety distance. Stage III: T1–4N1–2M0.

## Abbreviations

CRC, colorectal cancer; CTCs, circulating tumour cells; JHG, Jianpi Huatan granule; TCM, traditional Chinese medicine.

## References

[B1] Bray F. Ferlay J. Soerjomataram I. Siegel R. L. Torre L. A. Jemal A. (2018). Global cancer statistics 2018: GLOBOCAN estimates of incidence and mortality worldwide for 36 cancers in 185 countries. Ca. Cancer J. Clin. 68 (6), 394–424. 10.3322/caac.21492 30207593

[B2] Chen D. Yang Y. Yang P. (2019). Quxie capsule inhibits colon tumor growth partially through foxo1-mediated apoptosis and immune modulation. Integr. Cancer Ther. 18, 1534735419846377. 10.1177/1534735419846377 31030593 PMC6488785

[B3] Chen W. Zheng R. Baade P. D. Zhang S. Zeng H. Bray F. (2016). Cancer statistics in China, 2015. Ca. Cancer J. Clin. 66 (2), 115–132. 10.3322/caac.21338 26808342

[B4] Cheng X. Hu M. Chen C. Hou D. (2018). Computational analysis of mRNA expression profiles identifies a novel triple-biomarker model as prognostic predictor of stage II and III colorectal adenocarcinoma patients. Cancer Manag. Res. 10, 2945–2952. 10.2147/cmar.s170502 30214289 PMC6118290

[B5] Ching S. S. Y. Mok E. S. B. (2022). Adoption of healthy lifestyles among Chinese cancer survivors during the first five years after completion of treatment. Ethn. Health 27 (1), 137–156. 10.1080/13557858.2019.1634182 31238712

[B6] Guo Z. Jia X. Liu J. P. Liao J. Yang Y. (2012). Herbal medicines for advanced colorectal cancer. Cochrane Database Syst. Rev. 5, Cd004653. 10.1002/14651858.CD004653.pub2 22592697

[B7] Kugimiya N. Harada E. Suehiro Y. Suga A. Takemoto Y. Hamano K. (2019). Determination of thymidine phosphorylase expression level facilitates recurrence risk stratification in stage II/III colorectal cancer following adjuvant chemotherapy with oral fluoropyrimidines. Oncol. Lett. 17 (6), 5267–5274. 10.3892/ol.2019.10181 31186743 PMC6507397

[B8] Li M. Gu J. (2005). Changing patterns of colorectal cancer in China over a period of 20 years. World J. Gastroenterol. 11 (30), 4685–4688. 10.3748/wjg.v11.i30.4685 16094710 PMC4615411

[B9] Li S. T. Chi P. (2011). Evolution of the management of colorectal cancer using integrative medicine. Chin. J. Integr. Med. 17 (1), 73–79. 10.1007/s11655-011-0610-9 21258900

[B10] Lino-Silva L. S. Anchondo-Núñez P. Chit-Huerta A. Aguilar-Romero E. Morales-Soto J. Salazar-García J. A. (2019). Stage I-III colon cancer patients with tumor deposits behave similarly to stage IV patients. Cross-section analysis of 392 patients. J. Surg. Oncol. 120 (2), 300–307. 10.1002/jso.25482 31017669

[B11] Liu H. Liu H. Zhou Z. Parise R. A. Chu E. Schmitz J. C. (2018). Herbal formula Huang Qin Ge Gen Tang enhances 5-fluorouracil antitumor activity through modulation of the E2F1/TS pathway. Cell Commun. Signal. 16 (1), 7. 10.1186/s12964-018-0218-1 29458395 PMC5819251

[B12] Lv J. Jia Y. Li J. Kuai W. Li Y. Guo F. (2019). Gegen Qinlian decoction enhances the effect of PD-1 blockade in colorectal cancer with microsatellite stability by remodelling the gut microbiota and the tumour microenvironment. Cell Death Dis. 10 (6), 415. 10.1038/s41419-019-1638-6 31138779 PMC6538740

[B13] Miller K. D. Siegel R. L. Lin C. C. Mariotto A. B. Kramer J. L. Rowland J. H. (2016). Cancer treatment and survivorship statistics, 2016. Ca. Cancer J. Clin. 66 (4), 271–289. 10.3322/caac.21349 27253694

[B14] Munro A. Brown M. Niblock P. Steele R. Carey F. (2015). Do multidisciplinary team (mdt) processes influence survival in patients with colorectal cancer? A population-based experience. BMC Cancer 15, 686. 10.1186/s12885-015-1683-1 26463599 PMC4604766

[B15] Ntavatzikos A. Spathis A. Patapis P. Machairas N. Vourli G. Peros G. (2019). TYMS/KRAS/BRAF molecular profiling predicts survival following adjuvant chemotherapy in colorectal cancer. World J. Gastrointest. Oncol. 11 (7), 551–566. 10.4251/wjgo.v11.i7.551 31367274 PMC6657223

[B16] Oh H. J. Bae J. M. Wen X. Jung S. Kim Y. Kim K. J. (2019). p53 expression status is associated with cancer-specific survival in stage III and high-risk stage II colorectal cancer patients treated with oxaliplatin-based adjuvant chemotherapy. Br. J. Cancer 120 (8), 797–805. 10.1038/s41416-019-0429-2 30894685 PMC6474280

[B17] Ohlsson B. Pålsson B. (2003). Follow-up after colorectal cancer surgery. Acta Oncol. 42 (8), 816–826. 10.1080/02841860310019016 14968942

[B18] Pita-Fernández S. Alhayek-Aí M. González-Martín C. López-Calviño B. Seoane-Pillado T. Pértega-Díaz S. (2015). Intensive follow-up strategies improve outcomes in nonmetastatic colorectal cancer patients after curative surgery: A systematic review and meta-analysis. Ann. Oncol. 26 (4), 644–656. 10.1093/annonc/mdu543 25411419

[B19] Primrose J. N. Fuller A. Rose P. PereraSalazar R. Mellor J. Corkhill A. (2011). Follow-up after colorectal cancer surgery: Preliminary observational findings from the UK FACS trial. J. Clin. Oncol. 29 (15), 3521. 10.1200/jco.2011.29.15_suppl.3521

[B20] Rothé F. Maetens M. Rouas G. Paesmans M. Van den Eynde M. Van Laethem J. L. (2019). CTCs as a prognostic and predictive biomarker for stage II/III colon cancer: A companion study to the PePiTA trial. BMC Cancer 19 (1), 304. 10.1186/s12885-019-5528-1 30943928 PMC6446374

[B21] Siegel R. L. Jemal A. Wender R. C. Gansler T. Ma J. Brawley O. W. (2018). An assessment of progress in cancer control. Ca. Cancer J. Clin. 68 (5), 329–339. 10.3322/caac.21460 30191964

[B22] Siegel R. L. Miller K. D. Jemal A. (2019). Cancer statistics, 2019. Ca. Cancer J. Clin. 69 (1), 7–34. 10.3322/caac.21551 30620402

[B23] Snelgrove R. C. Subendran J. Jhaveri K. Thipphavong S. Cummings B. Brierley J. (2015). Effect of multidisciplinary cancer conference on treatment plan for patients with primary rectal cancer. Dis. Colon Rectum 58 (7), 653–658. 10.1097/dcr.0000000000000390 26200679

[B24] Sun L. Mao J. J. Vertosick E. Seluzicki C. Yang Y. (2018). Evaluating cancer patients' expectations and barriers toward traditional Chinese medicine utilization in China: A patient-support group-based cross-sectional survey. Integr. Cancer Ther. 17 (3), 885–893. 10.1177/1534735418777117 29888609 PMC6142069

[B25] Sun L. Yang Y. Vertosick E. Jo S. Sun G. Mao J. J. (2017). Do perceived needs affect willingness to use traditional Chinese medicine for survivorship care among Chinese cancer survivors? A cross-sectional survey. J. Glob. Oncol. 3 (6), 692–700. 10.1200/jgo.2016.007955 29244994 PMC5735974

[B26] Sun Q. He M. Zhang M. Zeng S. Chen L. Zhao H. (2021). Traditional Chinese medicine and colorectal cancer: Implications for drug discovery. Front. Pharmacol. 12, 685002. 10.3389/fphar.2021.685002 34276374 PMC8281679

[B27] Sung H. Ferlay J. Siegel R. L. Laversanne M. Soerjomataram I. Jemal A. (2021). Global cancer statistics 2020: GLOBOCAN estimates of incidence and mortality worldwide for 36 cancers in 185 countries. Ca. Cancer J. Clin. 71 (3), 209–249. 10.3322/caac.21660 33538338

[B28] Wang D. Yang Y. Jin L. Wang J. Zhao X. Wu G. (2019). Prognostic models based on postoperative circulating tumor cells can predict poor tumor recurrence-free survival in patients with stage II-III colorectal cancer. J. Cancer 10 (19), 4552–4563. 10.7150/jca.30512 31528219 PMC6746136

[B29] Wang W. Yin P. Liu Y. N. Liu J. M. Wang L. J. Qi J. L. (2021). Mortality and years of life lost of colorectal cancer in China, 2005-2020: Findings from the national mortality surveillance system. Chin. Med. J. 134 (16), 1933–1940. 10.1097/cm9.0000000000001625 34267069 PMC8382386

[B30] Winkels R. M. Kampman E. Wu M. (2021). Learning from East to West and vice versa: Clinical epidemiology of colorectal cancer in China. Cancer 127 (11), 1736–1738. 10.1002/cncr.33444 33788256

[B31] Xu Y. Mao J. J. Sun L. Yang L. Li J. Hao Y. (2017). Association between use of traditional Chinese medicine herbal therapy and survival outcomes in patients with stage II and III colorectal cancer: A multicenter prospective cohort study. J. Natl. Cancer Inst. Monogr. 2017 (52), lgx015. 10.1093/jncimonographs/lgx015 PMC6061258 29140496

[B32] Yang Y. F. Ge J. Z. Wu Y. Xu Y. Liang B. Y. Luo L. (2008). Cohort study on the effect of a combined treatment of traditional Chinese medicine and Western medicine on the relapse and metastasis of 222 patients with stage II and III colorectal cancer after radical operation. Chin. J. Integr. Med. 14 (4), 251–256. 10.1007/s11655-008-0251-9 19082795

[B33] You W. Sheng N. Yan L. Chen H. Gong J. He Z. (2019). The difference in prognosis of stage II and III colorectal cancer based on preoperative serum tumor markers. J. Cancer 10 (16), 3757–3766. 10.7150/jca.31660 31333793 PMC6636282

