# Supplementary material for: Efficacy of jianpi huatan granule in reducing colorectal cancer metastasis and recurrence after radical resection and adjuvant chemotherapy: Study protocol for a randomised, double-blind, placebo-controlled, multicentre trial
Source: Front Pharmacol. 2022 Sep 13;13:944475. doi: 10.3389/fphar.2022.944475 (PMC9513522; doi:10.3389/fphar.2022.944475)
Supplement: Supplementary file 2 [file DataSheet3.PDF]

Edmonton Symptom Assessment Scale（ESAS）

Please circle the number that best describes your average symptom over the past 24 hours

No Pain	0	1	2	3	4	5	6	7	8	9	10	Worst Pain
No Fatigue	0	1	2	3	4	5	6	7	8	9	10	Worst Fatigue
No Nausea	0	1	2	3	4	5	6	7	8	9	10	Worst Nausea
No Depressed	0	1	2	3	4	5	6	7	8	9	10	Worst Depressed
No Anxiety	0	1	2	3	4	5	6	7	8	9	10	Worst Anxiety
No Drowsiness	0	1	2	3	4	5	6	7	8	9	10	Worst Drowsiness
No Shortness of Breath	0	1	2	3	4	5	6	7	8	9	10	Worst Shortness of Breath
Best Appetite	0	1	2	3	4	5	6	7	8	9	10	Worst Appetite
Best Feeling or Well Being	0	1	2	3	4	5	6	7	8	9	10	Worst Feeling or Well Being
Best Sleep	0	1	2	3	4	5	6	7	8	9	10	Worst Sleep
