# Supplementary material for: Efficacy of jianpi huatan granule in reducing colorectal cancer metastasis and recurrence after radical resection and adjuvant chemotherapy: Study protocol for a randomised, double-blind, placebo-controlled, multicentre trial
Source: Front Pharmacol. 2022 Sep 13;13:944475. doi: 10.3389/fphar.2022.944475 (PMC9513522; doi:10.3389/fphar.2022.944475)
Supplement: Supplementary file 3 [file DataSheet1.PDF]

Traditional Chinese Medicine symptom scale

Symptoms	None	Mild	Moderate	Severe
1. Bloating	0□None	1□Mild abdominal distension	2□Abdominal distension and discomfort	3□Abdominal distension affects eating and rest
2. Fatigue	0□None	1□Fatigue, but can persist in physical labor	2□Fatigue, can barely insist on physical labor	3□Extreme fatigue, unable to stick to daily life
3. Pain	0□None	1□Occasionally, no medication needed	2□Pain longer, occasionally need medication to relieve	3□The pain is persistent, severe, and can only be relieved with medication
4. Dry stool	0□None	1□Dry stool, poor drainage, once every 1-2 days	2□Dry stools, difficult to discharge, once every 3-4 days	3□Stool hardened, dry and hard to resolve, once more than 4 days
5. Insomnia	0□None	1□Sleep time is slightly reduced	2□Occasionally insomnia	3□Unable to sleep
6. Loose stool	0□None	1□Occasionally have loose stools or have loose stools once a day	2□Loose stools 2-3 times a day	3□Loose stools more than 4 times a day
7. Poor appetite	0□None	1□Reduce food intake by 1/3	2□Reduce your food intake by 1/3 to 2/3	3□Reduce your food intake by more than two thirds
8. Sore waist and knees	0□None	1□Occasionally	2□Recurrent seizures	3□Persistent attacks, not easy to relieve
9. Stool irregular	0□None	1□Irregular bowel movements, no changes in traits	2□Stool irregular, sometimes dry and sometimes loose	3□Irregular bowel movements interfere with normal life
10. Shortness of breath	0□None	1□Shortness of breath after exercise	2□Shortness of breath after light exercise	3□Shortness of breath in a calm state
